# Parahippocampal gyrus expression of endothelial and insulin receptor signaling pathway genes is modulated by Alzheimer’s disease and normalized by treatment with anti-diabetic agents

**DOI:** 10.1371/journal.pone.0206547

**Published:** 2018-11-01

**Authors:** P. Katsel, P. Roussos, M. S. Beeri, M. A. Gama-Sosa, S. Gandy, S. Khan, V. Haroutunian

**Affiliations:** 1 Department of Psychiatry, Icahn School of Medicine at Mount Sinai, New York, New York, United States of America; 2 Department of Genetics and Genomic Science and Institute for Multiscale Biology, Icahn School of Medicine at Mount Sinai, New York, New York, United States of America; 3 Mental Illness Research, Education and Clinical Center, J.J. Peters VA Medical Center, Bronx, New York, United States of America; 4 The Joseph Sagol Neuroscience Center, Sheba Medical Center, Tel Aviv, Israel; 5 Department of Neuroscience, Icahn School of Medicine at Mount Sinai, New York, New York, United States of America; 6 Division of Neurology, J.J. Peters VA Medical Center, Bronx, New York, United States of America; 7 Department of Neurology, Icahn School of Medicine at Mount Sinai, New York, New York, United States of America; 8 General Medical Research Service, J.J. Peters VA Medical Center, Bronx, New York, United States of America; Nathan S Kline Institute, UNITED STATES

## Abstract

A large body of literature links risk of cognitive decline, mild cognitive impairment (MCI) and dementia with Type 2 Diabetes (T2D) or pre-diabetes. Accumulating evidence implicates a close relationship between the brain insulin receptor signaling pathway (IRSP) and the accumulation of amyloid beta and hyperphosphorylated and conformationally abnormal tau. We showed previously that the neuropathological features of Alzheimer’s disease (AD were reduced in patients with diabetes who were treated with insulin and oral antidiabetic medications. To understand better the neurobiological substrates of T2D and T2D medications in AD, we examined IRSP and endothelial cell markers in the parahippocampal gyrus of controls (N = 30), of persons with AD (N = 19), and of persons with AD and T2D, who, in turn, had been treated with anti-diabetic drugs (insulin and or oral agents; N = 34). We studied the gene expression of selected members of the IRSP and selective endothelial cell markers in bulk postmortem tissue from the parahippocampal gyrus and in endothelial cell enriched isolates from the same brain region. The results indicated that there are considerable abnormalities and reductions in gene expression (bulk tissue homogenates and endothelial cell isolates) in the parahippocampal gyri of persons with AD that map directly to genes associated with the microvasculature and the IRSP. Our results also showed that the numbers of abnormally expressed microvasculature and IRSP associated genes in diabetic AD donors who had been treated with anti-diabetic agents were reduced significantly. These findings suggest that anti-diabetic treatments may reduce or normalize compromised microvascular and IRSP functions in AD.

## Introduction

A significant body of literature links risk of cognitive decline, mild cognitive impairment (MCI), and dementia with Type 2 Diabetes (T2D) or pre-diabetes[1, 2]. T2D or impaired fasting glucose may be present in up to 80% of persons with Alzheimer’s disease (AD)[3]. Modifications in brain insulin metabolism are thought to be among the pathophysiological factors underlying dementia, whether due to AD[4] or to vascular cognitive impairment and dementia (VCID). Several studies suggest that pre-diabetes[5] and T2D may anticipate conversion to MCI[2, 6]. Imaging studies suggest significant changes in the brain microvasculature and in metabolic dysfunction[7] in persons with T2D, or even simple hyperglycemia in the absence of full blown diabetes, and dementia[8–10]. Accumulating evidence implicates a close relationship between the brain insulin receptor signaling pathway (IRSP) and the major neurobiological abnormalities of AD, amyloid beta (Aβ) and hyperphosphorylated and conformationally abnormal tau. Both pathologies have been shown to lower neuronal IR responses to insulin and to cause rapid and substantial loss of neuronal surface insulin receptors (IRs)[11]. Disruption of brain insulin signaling is one of the explanations for the consistently higher risk of AD and dementia in type 2 diabetic elderly[12]. Fat-feed laboratory transgenic mice models of AD that overexpress brain amyloidogenic genes develop glucose intolerance and insulin resistance, illustrating the potential bidirectional complexity of this relationship[13]. In AD patients, monotherapy with insulin[14] or with single representatives of other classes of hypoglycemic medications[15, 16] have been shown to not alter the risk of AD[17], but to potentially improve memory performance and slow cognitive decline. That cognitive impairment and AD neuropathology have been linked with T2D and even pre-diabetes suggests that the mechanisms underlying the relationship of T2D with dementia may be generalizable to non-T2D individuals. Although not performed on brain tissue, a recent study strongly supports an association between the molecular mechanisms of AD, insulin regulation, and T2D[18]. Integrative systems analysis of multiple tissues and organs in *ob/ob* mice identified APP as a top regulator of islet cell functions with the potential to regulate plasma insulin levels. This and other evidence for the complexity of the T2D-dementia interaction was comprehensively reviewed by Arnold and colleagues[19].

Recent evidence[20] suggests that metformin, and by extension other hypoglycemic medications[21], can significantly improve health- and lifespan. For example, metformin has the unique ability to correct the aging-related missorting of nuclear and cytoplasmic proteins[22]. In non-diabetic AD patients, monotherapy with insulin[23, 24] or with other hypoglycemic medications[14, 25] has shown some, albeit inconsistent, improvement in memory performance and slowing of AD symptom progression. In a series of studies[26, 27], our group showed that, when taken as a whole, T2D did not significantly affect the neuropathological sequelae of AD, but that the absence of a “T2D X AD neuropathology” interaction was apparently driven in large measure by the presence of antidiabetic drugs. Our studies showed that elderly persons with T2D treated with insulin *plus* other hypoglycemic agents (i.e., combination therapy) have dramatically less AD neuropathology (reduced densities of neuritic plaques and neurofibrillary tangles in the cortex) than otherwise similar non-T2D persons^2^. In an effort to understand better the neurobiological substrates of T2D and T2D medications in AD, we examined in the current study IRSP and endothelial cell markers in the parahippocampal gyri of persons with and without AD, T2D and T2D medications.

Insulin receptors (IR) are particularly abundant in brain regions supporting cognition, with recent evidence implicating a close relationship between the brain IR signaling pathway (IRSP) and the major neurobiological abnormalities of accumulations of Aβ and of conformationally abnormal and hyperphosphorylated tau (pTau)^e.g.,^[28]. *In vitro* studies^3-5^ provide support for our findings identifying the IRSP as an underlying mechanism by which combination of insulin with non-insulin T2D medications may modulate AD neuropathology even in the absence of comorbid T2D. Insulin binds to IR subunits (member of the receptor tyrosine kinase family engaging Akt and mTOR pathways). Phosphorylated insulin receptor substrate (IRS) scaffolding proteins link the IR to downstream signaling effectors including Akt. Akt activation inhibits pro-apoptotic signaling molecules such as BCL2-antagonist of cell death (BAD), B cell lymphoma 2 (BCL2), Forkhead Box O (FoxO), nuclear factor kappa B (NF-κB) and glycogen synthase kinase-3 beta (GSK3β). GSK3β by itself inhibits Akt by controlling mTORC, a key activating kinase for AKT[29]. GSK3β also phosphorylates β-catenin, targeting it for ubiquitination and proteasome dependent degradation[30]. Independent evidence implicates elements of this pathway in neuronal dysfunction, neurodegeneration and dementia[31–34]. In addition to this canonical “metabolic and anti-apoptotic” pathway of the IRSP, insulin binding to IR can also activate a second, SHC-ERK1/2, pro-survival pathway culminating in the activation of PPAR, and its coactivator PGC-1α, and interactions with the master cholesterol regulators RxR and LxR[35, 36]. Notably, these are the molecules that our studies indicate to be dysregulated in postmortem human AD brain[37, 38]. The IRSP in the brain contributes to the control of processes such as synaptic plasticity, e.g.,[27] [33, 34], neuroprotection, neurodegeneration, survival, growth, and energy metabolism, e.g.,[39], all of which are particularly relevant to cognition.

The mechanism by which insulin is delivered to the brain is uncertain, but insulin receptors (IR) on endothelial cells are the leading candidates[40]. Several non-mutually exclusive mechanisms may underlie the association between T2D and dementia, but increased cerebrovascular compromise and blood-brain barrier disruption[41–43]; defective signal transduction mechanism of central nervous system insulin receptors[44, 45]; and their potential interactions with amyloidogenic processes[44, 46–48] are among the most prominent ^see also^, [19]. Multiple studies have suggested that microvascular dysfunction, including permeability of the blood-brain barrier[49–52], may be a significant contributor to cognitive impairment in the elderly and in VCID or AD[53]. This evidence has come not only from numerous neuroimaging studies of microvascular dysfunction[54–56], but also from direct neuropathological investigations[57–65] and animal model systems[66, 67]. Similarly, microvascular damage and involvement, including dysfunction of endothelial cells in T2D, is undeniable[23, 55, 56, 68–71]. On the other hand, IRs in the brain do not appear to downregulate dramatically in response to high concentrations of insulin, and—in contrast to its role in glia and peripheral cells—insulin has a relatively limited role in regulating glucose metabolism in neurons[20, 72] and densities of IRs are not adversely affected in AD[47] when studied in bulk tissue assays. However, the vast majority of the studies of the brain IRSP in AD and T2D have been conducted in homogenized bulk tissue where even large changes and abnormalities in one or more cell types can be diluted or completely obscured when different cell types are intermixed in bulk-homogenate studies. In order to overcome this limitation and to address more directly the roles of T2D and antidiabetic medication in AD, we developed a method of endothelial cell enrichment from bulk human postmortem brain tissue and studied components of the IRSP and markers of endothelial cell function.

## Methods

### Tissue processing

This study involved analysis of postmortem human brain samples only. Consent for research use of the tissue was obtained from all donors. The collection and consent procedures were reviewed by the Mount Sinai (HS#: 13–00709 PS) and JJ Peters VA (ID01527) IRBs and were exempted from further review. The parahippocampal gyrus (Brodmann 36), was dissected from snap-frozen ~8 mm thick coronal sections. The dissected block was then pulverized using liquid nitrogen cooled mortar and pestle. The crushed homogenate was aliquoted into 50 and 100 mg aliquots. The general procedures for tissue acquisition, dissection and aliquot preparation have been described previously[73]. Some aliquots were used for bulk tissue analyses, whereas other sister aliquots from the same brain region of each donor were used for endothelial cell enrichment.

### Gene expression studies

Custom 51-plex QuantiGene assays (Life Technologies/Thermo, CA) were used for gene expression studies. Tissue and microvessel isolate homogenization, proteinase K treatment, probe hybridization and signal amplification were performed according to the manufacturer manual. Measurement of 51 genes (Table 1, and S1 File) was performed on Luminex 200 (Millipore, MA) instrument. Relative expression values were calculated using standard curve method and normalized to geometric means of four housekeeping genes included in the panel: *HMBS*, *NONO*, *PPIB and RPLP0*.

**Table 1:** Characteristics of the primary study cohorts.

	Mean PMI (Hours)	Mean CDR	Mean Age	Sex	Mean Cortical Plaques per mm^2	Mean Braak & Braak Score	Race (white; Black; Hispanic	Mean Blood glucose (mg/dl)
Control (N = 30)	12.85 (1.4)	0.43 (0.13)	83.17 (1.48)	14F; 16M	0.83 (0.32)	1.5 (0.32)	26; 3; 1	95.5 (22)
AD (N = 19)*	6.88 (1.17)	3.0 (0.28)	88.26 (2.01)	15F; 4M	9.58 (1.38)	4.89 (0.29)	17; 2; 1	119.4 (8)
AD+DM+Meds (N = 34)*	7.67 (0.98)	2.89 (0.26)	84.61 (1.49)	23F; 11M	8.39 (0.86)	4.64 (0.26)	26; 5; 3	148.8 (14)
Medication Characteristics of the AD+DM+Meds (N = 34 cohort)								
Medication Subset			Percent of Subset			Medication class		
Insulin Only			N = 15 (45%)			Insulin		
Oral Only			N = 12 (35%)			N = 9 (75%) Sulfonylurea N = 2 (17%) Metformin N = 1 (8%) Thiazolidinedione		
Insulin + Oral			N = 7 (21%)			N = 5 (79%) Sulfonylurea N = 2 (21%) Metformin		

* 17% (N = 9) of cases with AD (with or without DM medications) were treated with medications for AD (one was treated with memantine and 8 received donepezil) which were terminated at least 14 months prior to death.

### Selection of mRNA transcripts for expression studies

Twenty-four to twenty-six mRNA transcripts were selected for study. The selection of transcripts was based on several factors that included: known association with the IRSP; known and relatively enriched expression in endothelial cells[74], and relatively high or altered expression in bulk tissue microarray studies of AD[75, 76]. Twenty-three other transcripts associated with immune-inflammation, neurons, oligodendrocytes, astrocytes and microglia were also studied to survey other systems that may have been affected by T2D medications. The results of these later assays are shown in S1 File.

### Microvascular/endothelial cell enrichment

**Fig 1:**
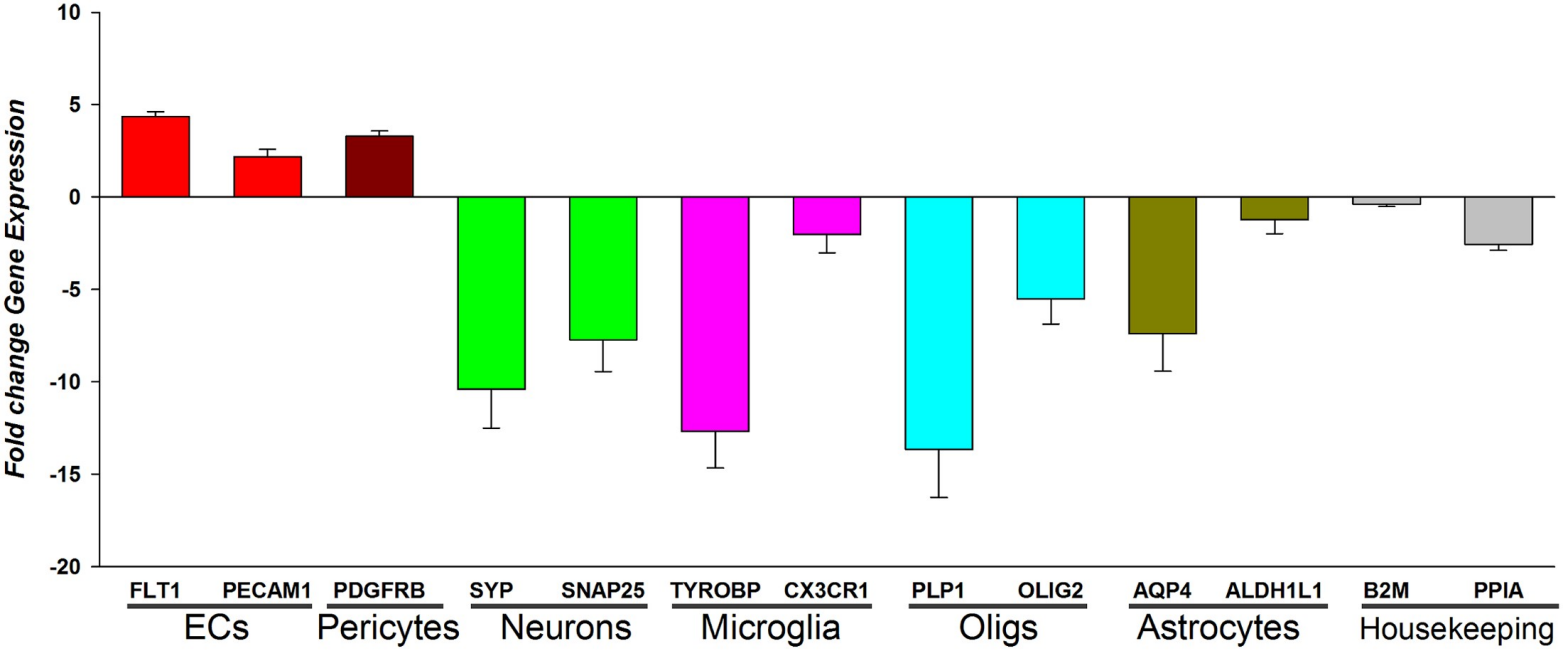
Enrichment of endothelial cell transcripts in microvascular isolates. The expression levels of selected endothelial cell markers and non-endothelial markers are expressed as fold change ratios relative to the levels of each transcript in bulk tissue homogenates.

One hundred milligrams of brain tissue was gently homogenized in cold 18% dextran/PBS containing protectRNA RNase inhibitor (Sigma, MO) (10 ml/g of tissue) using a Potter-Elvejehm with a loose-fit Teflon pestle (6–8 strokes at low speed)[77]. The homogenate was overlaid onto an equal volume of a discontinuous gradient of Ficoll-Paque PLUS^™^ and centrifuged for 30 min at 1,500 x g and 4°C. The resulting microvascular enriched pellet was resuspended and washed twice with PBS. The isolated microvascular/endothelial cell fragment was frozen and stored at -80C until further assays. In preliminary multiple gene expression studies, we have found this protocol to results in a significant (3–7 fold depending on the endothelial cell marker used) enrichment of microvascular/endothelial cell markers and a many-fold reduction in the levels of non-endothelial cell markers. Fig 1 shows one such example.

### Characteristics of brain tissue donors and molecular pathways of interest

The demographic characteristics and group stratification of study cases is shown in Table 1. All postmortem brain tissue donors were derived from the Mount Sinai NIH Neurobiobank (http://icahn.mssm.edu/research/nih-brain-tissue-repository/about). All brain tissue donations were derived from cases with written consent from the next of kin of each donor for research use. Each brain underwent a detailed neuropathological assessment as described previously[78, 79]. Donors were selected from over 1,900 cases for short postmortem interval (under 24 hours), high brain tissue pH (>6.0), the presence of either only AD pathology (as defined by CERAD[80]) or no evidence of neuropathology. Brodmann area 36 (the parahippocampal gyrus) was selected for study because transcriptome-wide analysis of 17 different brain regions showed it to be among the most transcriptionally vulnerable brain region in AD[76]. To enable direct comparison of assayed IRSP and endothelial cell mRNA expression, cases with AD and no known history of T2D were selected to match those with AD and a history of T2D as closely as possible with respect to both pre-agonal cognitive function and severity of neuropathology as defined by Braak score and density of cortical neuritic plaques. Cases with neuropathological evidence of non-AD pathology (e.g., Lewy body pathology, significant vascular pathology such as stroke or amyloid angiopathy, etc.) were excluded from all studies as described previously. Donors were included as presenting with T2D based on the American Diabetes Association criteria (symptoms of diabetes plus casual plasma glucose concentration > 200mg/dl; fasting plasma glucose > 126mg/dl; 2h plasma glucose>200mg/dl during OGTT); and/or record of receiving anti-diabetes medication; history of diabetes in the medical record. Diagnoses of T2D were ascertained from detailed structured review (>275 items) of all medical records and medical history. Similarly, medication use history and laboratory test results were derived from detailed reviews of medical records and semi-structured guided interviews of the next of kin or caregivers intimately acquainted with the donor.

### Statistical methods

Differential expression between disease status (control, AD) and treatment (insulin and/or oral anti-diabetes agents) was assessed using the limma package in R, with normalized gene expression matrix and the final covariate model, which included gender and PMI. Note that the analysis was run separately for gene expression derived from vessels and homogenate tissue. For each gene, least-squares linear regression was performed using limma to yield coefficients for the effect on gene expression of each variable on the right-hand side:

geneexpression∼Group+Gender+PMI;

where Group is defined based on combination of diagnosis and treatment [i.e., control, AD (no T2D, no T2D medications), and AD+T2D treated with insulin and/or oral anti-diabetes agents]. *P* values were adjusted for multiple hypothesis testing using false discovery rate (FDR) estimation, and the differentially expressed genes were determined as those with an estimated FDR ≤ 5%, or where specified FDR ≤ 7%.

## Results

The main analyses were performed on the expression of the genes shown in Table 2 with the primary goal of determining the extent to which IRSP and endothelial cell related transcripts were altered in AD vs. controls and whether these disease-associated changes were normalized in the brain of persons with AD receiving one or more antidiabetic medications of any kind and in any combination (i.e., insulin only, oral agents only, insulin plus oral agents).

**Table 2:** Abbreviations, brief function and cell-type expression and expression levels of IRSP and endothelial cell transcripts in AD-tissue and vessels.

Symbol	Description	System
*ANGPT1*	**Angiopoietin 1** is involved in vascular development and angiogenesis. http://web.stanford.edu/group/barres_lab/cgi-bin/geneSearch.py?geneNameIn=ANGPT1	Endothelial related
*CD59*	**CD59 glycoprotein**, aka **MAC-inhibitory protein** is mostly expressed in endothelial cells and oligodendrocytes. http://web.stanford.edu/group/barres_lab/cgi-bin/geneSearch.py?geneNameIn=CD59	Endothelial related
*CTNNB1*	**Catenin beta-1**, AKA **β-catenin**, is involved in regulation and coordination of cell–cell adhesion and gene transcription. It is a member of the Wnt signaling pathway. Highest expression is in endothelial cells. http://web.stanford.edu/group/barres_lab/cgi-bin/geneSearch.py?geneNameIn=CTNNB1	Endothelial related
*FLT1*	**Vascular endothelial growth factor receptor 1** shows tyrosine protein kinase activity that is important for the control of cell proliferation and differentiation. It is exclusively expressed in endothelial cells. http://web.stanford.edu/group/barres_lab/cgi-bin/geneSearch.py?geneNameIn=flt1	Endothelial related
*FOXF2*	**Forkhead box protein F2**. FOXF2 functions are not understood well, but it is expressed almost exclusively in endothelial cells. http://web.stanford.edu/group/barres_lab/cgi-bin/geneSearch.py?geneNameIn=FOXF2	Endothelial related
*ICAM1*	**Intercellular Adhesion Molecule 1** aka **D54** (**C**luster of **D**ifferentiation 54) encodes a cell surface glycoprotein which is expressed on endothelial cells and cells of the immune system. http://web.stanford.edu/group/barres_lab/cgi-bin/geneSearch.py?geneNameIn=ICAM1	Endothelial related
*IGF1R*	**Insulin-like growth factor 1 (IGF-1) receptor** belongs to the large class of tyrosine kinase receptors. This receptor mediates the effects of IGF-1, which is a polypeptide protein hormone similar in molecular structure to insulin. IGF-1 is highly expressed in endothelial cells. http://web.stanford.edu/group/barres_lab/cgi-bin/geneSearch.py?geneNameIn=IGF1R	Endothelial related
*PDGFRB*	**Beta-type platelet-derived growth factor receptor** Activation of PDGFRβ requires de-repression of the receptor’s kinase activity which is accomplished during PDGFRβ dimerization. Two of the five PDGF isoforms activate PDGFRβ (PDGF-B and PDGF-D) which phosphorylates itself and other proteins, and engages intracellular signaling pathways associated with migration and proliferation. PDGFRβ is mostly expressed in astrocytes and endothelial cells. http://web.stanford.edu/group/barres_lab/cgi-bin/geneSearch.py?geneNameIn=PDGFRB	Endothelial related
*PECAM1*	**Platelet endothelial cell adhesion molecule** aka CD31, plays a key role in removing aged neutrophils from the body. PECAM-1 is found on the surface of platelets, monocytes, neutrophils, and some types of T-cells, and makes up a large portion of endothelial cell intercellular junctions. The encoded protein is a member of the immunoglobulin superfamily, it is expressed almost exclusively on endothelial cells and is likely involved in leukocyte transmigration, angiogenesis, and integrin activation. http://web.stanford.edu/group/barres_lab/cgi-bin/geneSearch.py?geneNameIn=PECAM1	Endothelial related
*SLC2A1*	**Glucose transporter 1** (or **GLUT1**), aka **solute carrier family 2, facilitated glucose transporter member 1** is expressed mainly in endothelial cells. http://web.stanford.edu/group/barres_lab/cgi-bin/geneSearch.py?geneNameIn=SLC2A1	Endothelial related
*VCAM1*	**Vascular cell adhesion protein 1** is expressed on both large and small blood vessels only after the endothelial cells are stimulated by cytokines. The VCAM-1 protein mediates the adhesion of lymphocytes, monocytes, eosinophils, and basophils to the vascular endothelium. Upregulation of VCAM-1 in endothelial cells by cytokines occurs as a result of increased gene transcription (e.g., in response to Tumor necrosis factor-alpha (TNF-α) and Interleukin-1 (IL-1)). VCAM-1 protein is an endothelial ligand for VLA-4 (Very Late Antigen-4 or integrin α4β1) of the β1 subfamily of integrins. It is almost exclusively expressed in astroglial. http://web.stanford.edu/group/barres_lab/cgi-bin/geneSearch.py?geneNameIn=VCAM1	Endothelial related
*VEGFA*	**Vascular endothelial growth factor A** is a member of the platelet-derived growth factor (PDGF) family. It acts on endothelial cells to increase permeability. It is expressed in mostly in astrocytes but also in neurons and OPCs. http://web.stanford.edu/group/barres_lab/cgi-bin/geneSearch.py?geneNameIn=VEGFA	Endothelial related
*VWF*	**Von Willebrand factor** is expressed mostly in endothelial cells. http://web.stanford.edu/group/barres_lab/cgi-bin/geneSearch.py?geneNameIn=VWF	Endothelial related

*AKT1*	**V-akt murine thymoma viral oncogene homolog 1; AKT1** gene encodes an enzyme in the serine/threonine kinase family and is a key signaling molecule in the IRSP. http://web.stanford.edu/group/barres_lab/cgi-bin/geneSearch.py?geneNameIn=AKT1	IRSP related
*AKT3*	**v-akt murine thymoma viral oncogene homolog 3; AKT3**, regulates cell signaling in response to insulin and growth factors. http://web.stanford.edu/group/barres_lab/cgi-bin/geneSearch.py?geneNameIn=AKT3	IRSP related
*DDIT4*	**DNA-damage-inducible transcript 4 protein** (**DDIT4**) acts as a negative regulator of mTOR. Metformin increases DDIT4 expression. http://web.stanford.edu/group/barres_lab/cgi-bin/geneSearch.py?geneNameIn=DDIT4	IRSP related
*FTO*	**Fat mass and obesity-associated protein**. http://web.stanford.edu/group/barres_lab/cgi-bin/geneSearch.py?geneNameIn=FTO	IRSP related
*GSK3B*	**Glycogen synthase kinase 3 beta**, is a kinase and part of the IRSP. http://web.stanford.edu/group/barres_lab/cgi-bin/geneSearch.py?geneNameIn=GSK3B	IRSP related
*INSR*	**Insulin receptor** (**IR**) is an insulin, IGF-I and IGF-II activated transmembrane receptor that is expressed in most cells. http://web.stanford.edu/group/barres_lab/cgi-bin/geneSearch.py?geneNameIn=INSR	IRSP related
*IRS1*	**Insulin receptor substrate** participates in transmitting signals from the insulin and insulin-like growth factor-1 (IGF-1) receptors to intracellular pathways PI3K / Akt and Erk MAP kinase pathways. http://web.stanford.edu/group/barres_lab/cgi-bin/geneSearch.py?geneNameIn=IRS1	IRSP related
*IRS2*	**Insulin receptor substrate 2** is a cytoplasmic signaling molecule that mediates effects of insulin, insulin-like growth factor 1, and some cytokines by acting as a molecular adaptor between diverse receptor tyrosine kinases. http://web.stanford.edu/group/barres_lab/cgi-bin/geneSearch.py?geneNameIn=irs2	IRSP related
*MTOR*	**mechanistic target of** rapamycin (**mTOR**), (formerly **mammalian target of rapamycin**) is a serine/threonine kinase and is part of the IRSP. http://web.stanford.edu/group/barres_lab/cgi-bin/geneSearch.py?geneNameIn=MTOR	IRSP related
*PPARGC1A*	**Peroxisome proliferator-activated receptor gamma coactivator 1-alpha** regulates genes associated with energy metabolism. PPARGC1A interacts with the nuclear receptor PPAR-γ and is a downstream member of the IRSP. It is expressed in astrocytes, neurons and oligodendrocytes. http://web.stanford.edu/group/barres_lab/cgi-bin/geneSearch.py?geneNameIn=PPARGC1A	IRSP related
*RICTOR*	**Rapamycin-insensitive companion of mammalian target of rapamycin** (**RICTOR**) is a subunit of mTOR and part of the IRSP. http://web.stanford.edu/group/barres_lab/cgi-bin/geneSearch.py?geneNameIn=rictor	IRSP related
*RPS6KB1*	**Ribosomal protein S6 kinase beta-1 (S6K1)**, aka **p70S6 kinase** (**p70S6K**, **p70-S6K**), is a protein kinase that phosphorylates threonine 389 and activates mTOR and correlated with autophagy inhibition. The kinase activity of RPS6KB1 protein leads to an increase in protein synthesis and cell proliferation. It is expressed in most cell types. http://web.stanford.edu/group/barres_lab/cgi-bin/geneSearch.py?geneNameIn=RPS6KB1	IRSP related
*RPTOR*	**Regulatory-associated protein of mTOR** AKA **KIAA1303** encodes part of a signaling pathway regulating cell growth responding to nutrient and insulin levels. http://web.stanford.edu/group/barres_lab/cgi-bin/geneSearch.py?geneNameIn=RPTOR	IRSP related
*SLC2A4*	**Glucose transporter type 4**, aka **GLUT4** is the insulin-regulated glucose transporter found primarily in adipose tissues and striated muscle (skeletal and cardiac). It is expressed in most cell types, but especially in astrocytes. http://web.stanford.edu/group/barres_lab/cgi-bin/geneSearch.py?geneNameIn=SLC2A4	IRSP related
*TBC1D4*	**TBC1 domain family member 4** now known as **AS160** encodes Rba GTPase-activating protein and is a substrate for Akt2. http://web.stanford.edu/group/barres_lab/cgi-bin/geneSearch.py?geneNameIn=TBC1D4	IRSP related

### Altered mRNA expression in whole tissue homogenates of the parahippocampal gyrus in AD and anti-T2D medicated AD

**Fig 2:**
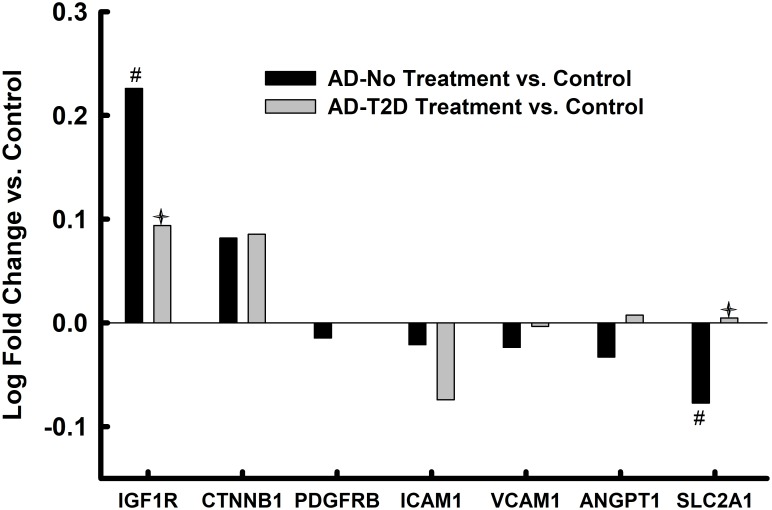
Endothelial cell markers in parahippocampal gyrus bulk tissue. Values represent relative log fold change in persons with AD relative to controls and log fold change in persons with AD and T2D who had been treated with anti-diabetes agents. * = p<0.05 after FDR correction; # = p<0.05 without FDR correction; ✦ = p<0.05 AD-No Treatment vs. AD-T2D Treatment.

**Fig 3:**
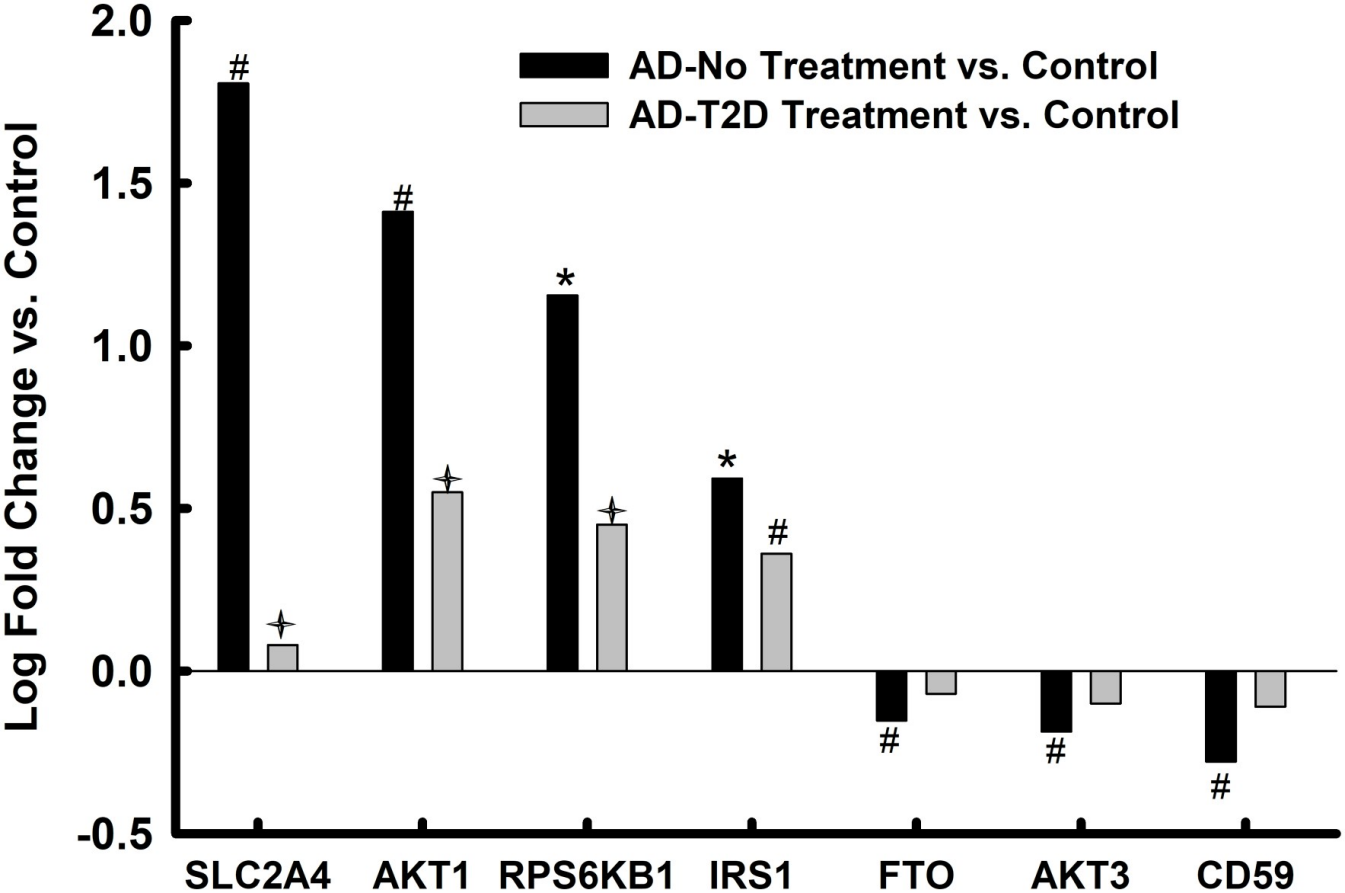
IRSP-associated markers in parahippocampal gyrus bulk tissue. Values represent relative log fold change in persons with AD relative to controls and log fold change in in persons with AD and T2D who had been treated with anti-diabetes agents. * = p<0.05 after FDR correction; # = p<0.05 without FDR correction; ✦ = p<0.05 AD-No Treatment vs. AD-T2D Treatment.

Compared to controls, 12 of the 18-endothelial cell and IRSP-associated genes assayed were significantly (12 of 18 unadjusted ps<0.05; 6 of 12 after FDR correction) altered in AD (Figs 2 and 3). One third of the altered genes (unadjusted ps<0.05), or 44% after FDR correction, were genes with preponderant expression in endothelial cells[74]. In AD donors who had a history of receiving antidiabetic treatment, only 4 of these 12 genes remained significant before FDR correction and only 1 (*ANGPT1*) after FDR correction.

### Altered mRNA expression in endothelial cells of the parahippocampal gyrus in AD and anti-T2D medicated AD

**Fig 4:**
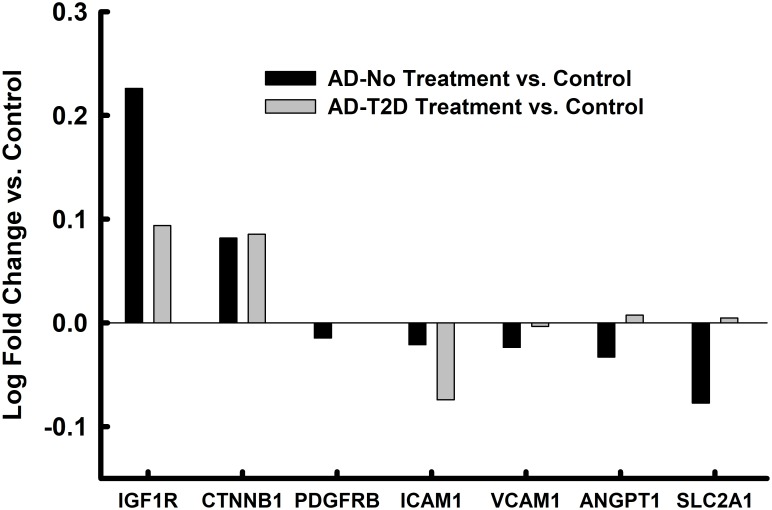
Endothelial cell markers in microvascular enriched isolates from the parahippocampal gyrus. Values represent relative log fold change in persons with AD relative to controls and log fold change in persons with AD and T2D who had been treated with anti-diabetes agents. * = p<0.05 after FDR correction; # = p<0.05 without FDR correction; ✦ = p<0.05 AD-No Treatment vs. AD-T2D Treatment.

**Fig 5:**
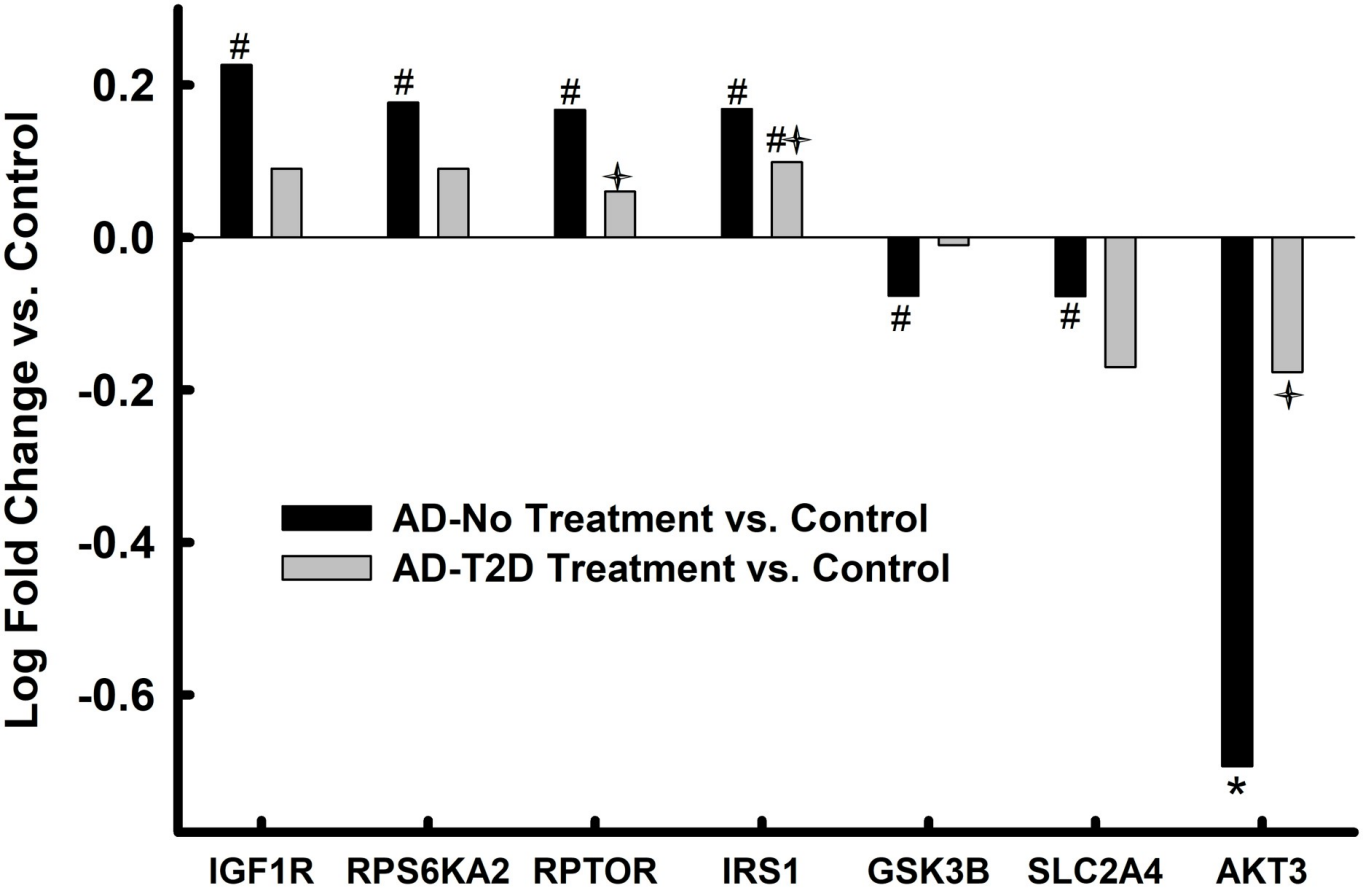
IRSP-associated markers in microvascular enriched isolates from the parahippocampal gyrus. Values represent relative log fold change in persons with AD relative to controls and log fold change in persons with AD and T2D who had been treated with anti-diabetes agents. * = p<0.05 after FDR correction; # = p<0.05 without FDR correction; ✦ = p<0.05 AD-No Treatment vs. AD-T2D Treatment.

Compared to EC enriched fraction of controls, 5 (unadjusted for FDR) endothelial and IRSP-related genes were abnormally expressed in untreated persons with AD (Figs 4 and 5). None of the IRSP/endothelial cell associated genes, except for AKT3, met the FDR corrected threshold. Only one of these 5 genes (*IRS1*) remained significantly different in expression in the endothelial cell fraction of treated persons with AD, and even the expression levels of this gene approached that of the controls, nominally. Interestingly, the expression level of IRS1 was not changed in the endothelial fractions derived from persons with AD, but its expression increased significantly (corrected p = 0.05) in those AD donors who had been treated with antidiabetic medications.

### Non-IRSP and/or non-endothelial cell mRNAs in the parahippocampal gyrus in AD and anti-T2D medicated AD

Twenty-three mRNAs representing neuronal, oligodendroglial, astrocytic, microglial cell types, and cell-cell adhesion, inflammation/immune, cell fate markers were also selected (S1 File) to study the consequences of antidiabetic treatment on some of the more non-IRSP/endothelial cell functions of the parahippocampal gyrus. Not surprisingly, the expression of 8 of the 23 mRNAs studied was affected in the whole tissue homogenates of untreated persons with AD and included mRNAs encoding for proteins associated with synaptic function, astrocytes, cell adhesion and immune/inflammation responses. Treatment with antidiabetic medications reduced the number of adversely affected mRNAs to 3 that included markers of immune/inflammation and cell-cell adhesion. Significantly fewer of these markers were affected in the endothelial cell enriched fraction, where 2 (oligodendroglial and nuclear RNA retention) of the 23 markers were affected in untreated AD samples and altered expression remained significant, albeit nominally moderated in the treatment group.

## Discussion

Numerous clinical, animal model, and postmortem studies have suggested that treatment of persons suffering from dementia with antidiabetic medications may have beneficial effects on cognitive function and on AD-related neuropathology[19, 26]. A number of recent studies and reviews have drawn attention to the role(s) of the brain microvasculature in dementia[56] and especially in persons with diabetes[55, 56, 81]. The current study was designed to uncover some of the transcriptomic substrates for microvascular abnormalities in AD and to delineate whether any beneficial effects of T2D medications could be attributed to the restoration of microvascular and endothelial cell attributes affected in AD.

The results indicated that there are considerable abnormalities and reductions in gene expression (whole tissue homogenates and endothelial cell isolates) in the parahippocampal gyrus of persons with AD that map directly to genes associated with the microvasculature and the IRSP. Whether these endothelial and IRSP abnormalities contribute to the genesis of AD neuropathology, or whether they result from other neuropathological changes cannot be determined in postmortem studies. The findings also showed that the numbers of abnormally expressed microvasculature and IRSP associated genes in diabetic AD donors who had been treated with anti-diabetic agents were reduced significantly. Of course, it can be argued that in the absence of untreated studied cases with AD and T2D, the “normalization” of microvasculature and the IRSP gene expression could be due to T2D and not its treatment. Although this argument cannot be countered by the results of this study, we find this alternative explanation to be less biologically plausible given knowledge of the well-documented detrimental effects of T2D on brain parenchyma and especially on the brain microvasculature. The interpretation that treatment of T2D “normalized” multiple transcriptional abnormalities associated with AD is, however, consistent with the results of some of the clinical trials reported to date[24, 82] as well as our earlier studies of the effects of anti-diabetic treatments on AD neuropathology[26, 27]. That AKT3 expression was dramatically and robustly reduced in endothelial cells, and to a lesser extent in whole tissue homogenates suggests that tissue level expression was driven by changes in the endothelial transcriptome and that the disruption of components of the IRSP in endothelial cells is a significant participant in AD neuropathology.

The expression levels of *GLUT4* (*SLC2A4*), which encodes for an insulin-regulated glucose transporter, were significantly upregulated in bulk tissue from the parahippocampal gyrus of AD donors (Fig 3), while the expression levels of this same transcript were significantly reduced in the endothelial cell enriched isolates derived from the same donors and brain region. On the other hand, the expression levels of *GLUT4* in persons with AD and T2D who had been treated with anti-diabetic medications were similar to the levels detected in controls. This suggests that anti-diabetic medications restored homeostasis to this critical glucose transporter and that dis-homeostasis of glucose transport in brain endothelial and non-endothelial cells may be a critical abnormality in AD that is restored by anti-diabetes medications. Animal model and *in vitro* studies will need to be conducted to determine whether dysregulation of *GLUT4* may be a key contributor to at least some of the other IRSP and endothelial cell abnormalities observed in the current study. Zlokovic and colleagues have independently implicated GLUT1, another glucose transporter, as a key player in the microvascular pathology associated with AD[83].

“Normalization” of abnormally expressed genes in AD by anti-T2D treatment was not limited to transcripts associated with the IRSP and endothelial cells, but as shown in Figs 1 and 2 of the S1 File carried over to genes associated with immune-inflammation, microglia, cell-adhesion and synaptic function. These findings are not surprising if it is assumed that treatment with anti-T2D medications results in decreased overall inflammation and improved insulin signaling.

The fact that no brains from AD+T2D diabetic donors that had not been treated with antidiabetic agents were included in this study is a distinct weakness and detracts from interpretative power. However, in developed countries, the vast majority of persons diagnosed with T2D receive insulin or oral anti-diabetes medications, making it difficult to include such a group in clinical and postmortem studies. In addition, interpretation of results, even if such a group had been included, would have been hampered since, almost by definition, the severity of T2D would have been significantly less than that of the treated subjects. Despite the imperfect nature of mouse models, these variables will be easier to control in such a system, and we plan to attempt to address some of these questions left open in our postmortem human studies by employing mouse models of amyloidosis or tauopathy, each of which will be studied without or with diabetes, and each of those groups, in turn, can be studied with or without antidiabetic medication. Choice of the adequate mouse model of diabetes will be key since we have observed some unexpected effects in some transgenic mouse models for amyloidosis[84] and the effect of a deficiency of the T2D gene, *Sorcs1*[85]. Finally, we might also need to examine the effects of young ages vs older as additional parameters in this highly ambitious study.

As noted in Table 1, the great majority of the donors (79+%) who were treated with oral anti-diabetes agents were treated with sulfonylureas. Although this makes it easier to attribute any oral medication effects to sulfonylureas, it detracts from our ability to distinguish between the effects of the myriad of different agents with different mechanisms of action. The inability to discern the specific neuro/vascular effects of different anti-diabetic agents in this study also precluded our ability to assess how the reported interactions of metformin and APP/Aβ influence IRSP and endothelial cell markers[86–88]. In addition, it would have been of significant interest to stratify the studied cohort according to whether they received insulin only, oral agents only, or insulin plus oral agents as well as the efficacy and duration of treatment. Unfortunately, extended medical histories (e.g., since mid-life) were not available since the study cohort was nursing home based and the sample size was not large enough for adequate statistical power to analyze the results with medication type granularity.

Challenge notwithstanding, about half of diabetics develop dementia and that roughly doubles the cost of caring for each demented diabetic patient especially since their cognitive decline makes it impossible for the patient to participate in the monitoring and modulation of his/her diabetic status. Given the dual epidemics of T2D and dementia in the most rapidly growing segment of our population, there is enormous importance in elucidating the basis for cognitive impairment in T2D so that potentially meaningful interventions can be evaluated.

## Supporting information

S1 File Other transcripts. (DOCX)

S2 File Original raw data. (XLSX)

S1 Fig Significantly affected non-endothelial and non-IRSP associated transcripts in parahippocampal gyrus bulk tissue. Values represent relative log fold change in persons with AD relative to controls and log fold change in persons with AD and T2D who had been treated with anti-diabetes agents. * = p<0.05 after FDC correction; # = p<0.05 without FDR correction.
(TIF)

S2 Fig Significantly affected non-endothelial and non-IRSP associated transcripts in microvascular enriched isolates from the parahippocampal gyrus. Values represent relative log fold change in persons with AD relative to controls and log fold change in persons with AD and T2D who had been treated with anti-diabetes agents. * = p<0.05 after FDC correction; # = p<0.05 without FDR correction.
(TIF)
