# Supplementary material for: Parahippocampal gyrus expression of endothelial and insulin receptor signaling pathway genes is modulated by Alzheimer’s disease and normalized by treatment with anti-diabetic agents
Source: PLoS One. 2018 Nov 1;13(11):e0206547. doi: 10.1371/journal.pone.0206547 (PMC6211704; doi:10.1371/journal.pone.0206547)
Supplement: S1 File — (DOCX) [file pone.0206547.s001.docx]

| S1 Table 1. Abbreviations, brief function and cell-type expression of non-IRSP and non-endothelial cell transcripts in AD-tissue and vessels. | | |
| --- | --- | --- |
| Symbol | Description | System |
| ADAMTS1 | **A disintegrin and metalloproteinase with thrombospondin motifs 1** expression may be associated with inflammatory processes. <http://web.stanford.edu/group/barres_lab/cgi-bin/geneSearch.py?geneNameIn=ADAMTS1> | Most cells |
| ALDH1L1 | Codes for the enzyme **10-formyltetrahydrofolate dehydrogenase** which is a member of the aldehyde dehydrogenase family and is exclusively expressed by astrocytes. [http://web.stanford.edu/group/barres_lab/cgi-bin/geneSearch.py?geneNameIn=aldh1l1](%20http:/web.stanford.edu/group/barres_lab/cgi-bin/geneSearch.py?geneNameIn=aldh1l1) | Astrocytes |
| APOLD1 | **Apolipoprotein L domain containing 1** is an early response protein involved in the regulation of cell signaling and vascular function. <http://web.stanford.edu/group/barres_lab/cgi-bin/geneSearch.py?geneNameIn=apold1> | Most cells |
| AQP4 | **Aquaporin 4** is associated with water regulation in astrocytes. <http://web.stanford.edu/group/barres_lab/cgi-bin/geneSearch.py?geneNameIn=apold1> | Most cells |
| CD163 | **Cluster of Differentiation 163** is the high affinity scavenger receptor for the hemoglobin-haptoglobin complex. <http://web.stanford.edu/group/barres_lab/cgi-bin/geneSearch.py?geneNameIn=aqp4> | Astrocytes |
| CEBPD | **CCAAT/enhancer-binding protein delta** The encoded protein is important in the regulation of genes involved in immune and inflammatory responses, and the regulation of genes associated with activation and/or differentiation of macrophages and the regulation of apoptosis and call proliferation. <http://web.stanford.edu/group/barres_lab/cgi-bin/geneSearch.py?geneNameIn=cebpd> | Microglia and others |
| CX3CR1 | **CX3C chemokine receptor 1** (CX3CR1) aka **fractalkine receptor** is a transmembrane protein and chemokine involved in the migration of microglia in the developing CNS. <http://web.stanford.edu/group/barres_lab/cgi-bin/geneSearch.py?geneNameIn=cx3cr1> | Microglia |
| FCGR1A | **High affinity immunoglobulin gamma Fc receptor I** is expressed almost exclusively in microglia. <http://web.stanford.edu/group/barres_lab/cgi-bin/geneSearch.py?geneNameIn=fcgr1a> | microglia |
| HILPDA | Hypoxia-inducible lipid droplet-associated protein. <http://web.stanford.edu/group/barres_lab/cgi-bin/geneSearch.py?geneNameIn=Hilpda> | Astrocytes and endothelial cells |
| HMBS | **Porphobilinogen deaminase** (hydroxymethylbilane synthase, or uroporphyrinogen I synthase) is involved in the heme biosynthetic pathway. <http://web.stanford.edu/group/barres_lab/cgi-bin/geneSearch.py?geneNameIn=hmbs> | Most cells |
| ITGA5 | **Integrin alpha-5** participates in adhesion and cell-surface mediated signaling. Expressed in microglia and endothelial cells, and also, but at lower levels in astrocytes. <http://web.stanford.edu/group/barres_lab/cgi-bin/geneSearch.py?geneNameIn=itga5> | Microglia |
| ITGB1 | **Integrin beta-1** also known as **CD29** is involved in cell adhesion and embryogenesis, hemostasis, tissue repair and immune responses. <http://web.stanford.edu/group/barres_lab/cgi-bin/geneSearch.py?geneNameIn=itgb1> | Most cells |
| NCAM1 | **Neural cell adhesion molecule** (**NCAM**), also called **CD56**, is a homophylic binding glycoprotein expressed on the surface of neurons, glia, skeletal muscle and natural killer cells. NCAM has a role in cell–cell adhesion, neurite outgrowth, synaptic plasticity, and learning and memory. <http://web.stanford.edu/group/barres_lab/cgi-bin/geneSearch.py?geneNameIn=ncam1> | Most cells |
| NEUROD2 | **Neurogenic differentiation factor 2** is a helix-loop-helix (bHLH) protein that induces transcription from neuron-specific promoters, such as the GAP-43 promoter and plays a role in maintenance of neuronal cell fates. <http://web.stanford.edu/group/barres_lab/cgi-bin/geneSearch.py?geneNameIn=neurod2> | Astrocytes / neurons |
| NONO | **Non-POU domain-containing octamer-binding protein** (NonO) may be part of the process in the nucleus that is responsible for the retention of RNAs that are defective, not yet mature enough to be exported or are designed to be retained in nucleus. <http://web.stanford.edu/group/barres_lab/cgi-bin/geneSearch.py?geneNameIn=nono> | Most cells |
| PLP1 | **Proteolipid protein 1** (PLP1) is a form of myelin proteolipid protein (PLP) expressed exclusively in oligodendrocytes. <http://web.stanford.edu/group/barres_lab/cgi-bin/geneSearch.py?geneNameIn=plp1> | Oligodendrocytes |
| PPIB | **Peptidyl-prolyl cis-trans isomerase B** is a chaperonin/cyclophilin that regulates protein folding and participates in biological processes such as ischemic reperfusion injury. <http://web.stanford.edu/group/barres_lab/cgi-bin/geneSearch.py?geneNameIn=ppib> | Most cells |
| PPIH | **Peptidyl-prolyl cis-trans isomerase H** This protein possess PPIase activity and may act as a protein chaperone that mediates the interactions between different proteins inside the spliceosome. It is expressed everywhere. <http://web.stanford.edu/group/barres_lab/cgi-bin/geneSearch.py?geneNameIn=ppih> | Neurons and others |
| PTEN | **Phosphatase and tensin homolog** acts as a phosphatase to dephosphorylate PIP_3_. PTEN catalyses the dephosphorylation of the 3` phosphate of the inositol ring in PIP_3_, resulting in the biphosphate product PIP_2_ (PtdIns(4,5) P2). This dephosphorylation results in inhibition of the [AKT](https://en.wikipedia.org/wiki/AKT) signaling pathway. <http://web.stanford.edu/group/barres_lab/cgi-bin/geneSearch.py?geneNameIn=pten> | Most cells |
| RPLP0 | **60S acidic ribosomal protein P0**. Housekeeping gene. <http://web.stanford.edu/group/barres_lab/cgi-bin/geneSearch.py?geneNameIn=RPLP0> | All cells |
| RPS6KA2 | **Ribosomal protein S6 kinase alpha-2** The activity of this protein is implicated in controlling cell growth and differentiation. <http://web.stanford.edu/group/barres_lab/cgi-bin/geneSearch.py?geneNameIn=rps6ka2n> | Most cells |
| SNAP25 | **Synaptosomal-associated protein 25** is responsible for forming a tight complex that brings the synaptic vesicle and plasma membranes together. http://web.stanford.edu/group/barres_lab/cgi-bin/geneSearch.py?geneNameIn=span25 | Neurons |
| SOX10 | **Transcription factor SOX-10** encodes a member of the SOX (SRY-related HMG-box) family of transcription factors involved in the regulation of oligodendrocytes and embryonic development and determination of cell fate. <http://web.stanford.edu/group/barres_lab/cgi-bin/geneSearch.py?geneNameIn=sox10> | Oligodendrocytes |

S1 Figure 1. Significantly affected non-endothelial and non-IRSP associated transcripts in parahippocampal gyrus bulk tissue. Values represent relative log fold change in persons with AD relative to controls and log fold change in persons with AD and T2D who had been treated with anti-diabetes agents. *= p<0.05 after FDC correction; #= p<0.05 without FDR correction.

S1 Figure 2. Significantly affected non-endothelial and non-IRSP associated transcripts in microvascular enriched isolates from the parahippocampal gyrus. Values represent relative log fold change in persons with AD relative to controls and log fold change in persons with AD and T2D who had been treated with anti-diabetes agents. *= p<0.05 after FDC correction; #= p<0.05 without FDR correction.
