# Supplementary figures and images for: Parahippocampal gyrus expression of endothelial and insulin receptor signaling pathway genes is modulated by Alzheimer’s disease and normalized by treatment with anti-diabetic agents
Source: PLoS One. 2018 Nov 1;13(11):e0206547. doi: 10.1371/journal.pone.0206547 (PMC6211704; doi:10.1371/journal.pone.0206547)

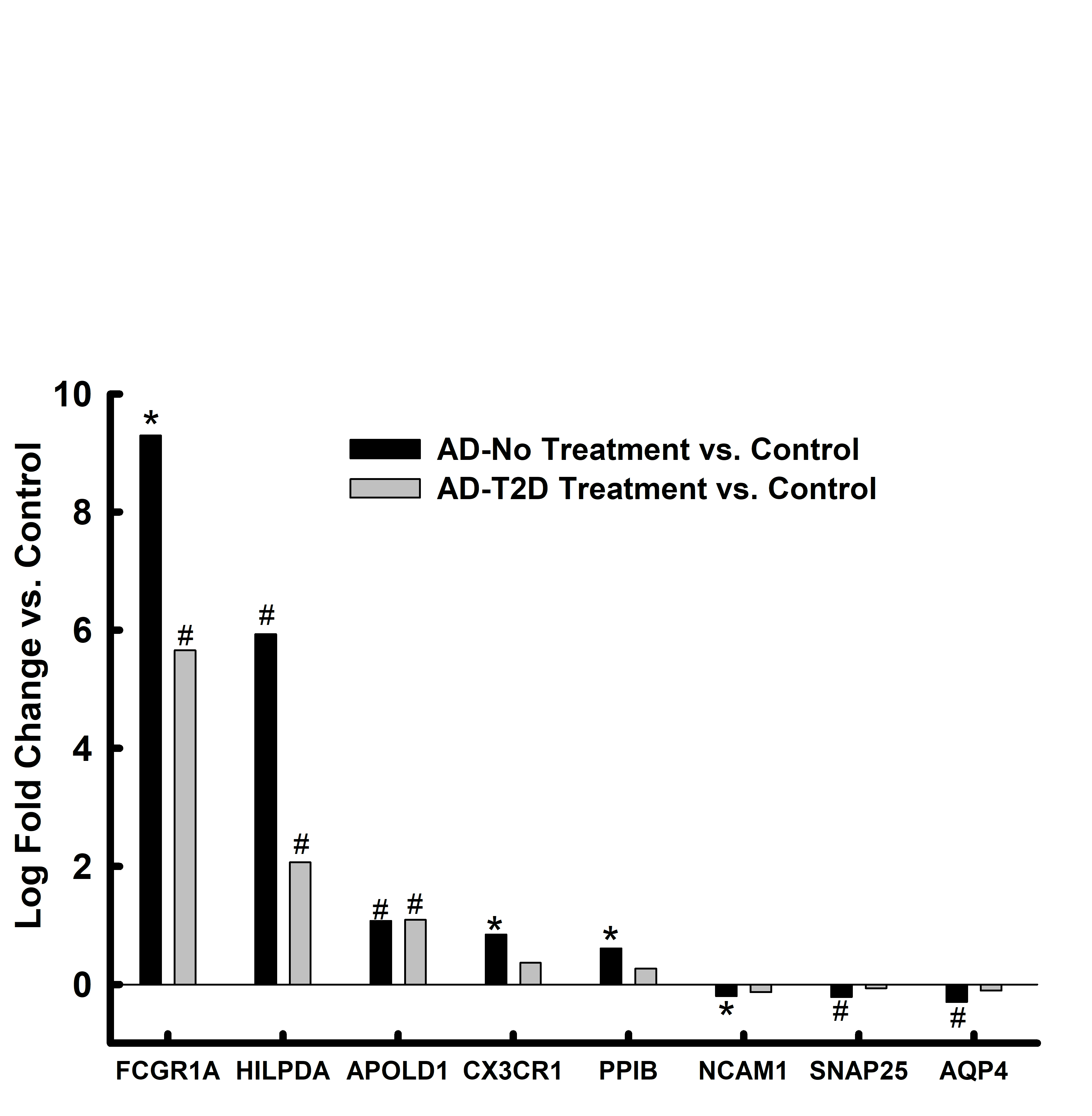

Supplement: S1 Fig — Values represent relative log fold change in persons with AD relative to controls and log fold change in persons with AD and T2D who had been treated with anti-diabetes agents. * = p<0.05 after FDC correction; # = p<0.05 without FDR correction. (TIF) [file pone.0206547.s003.TIF]

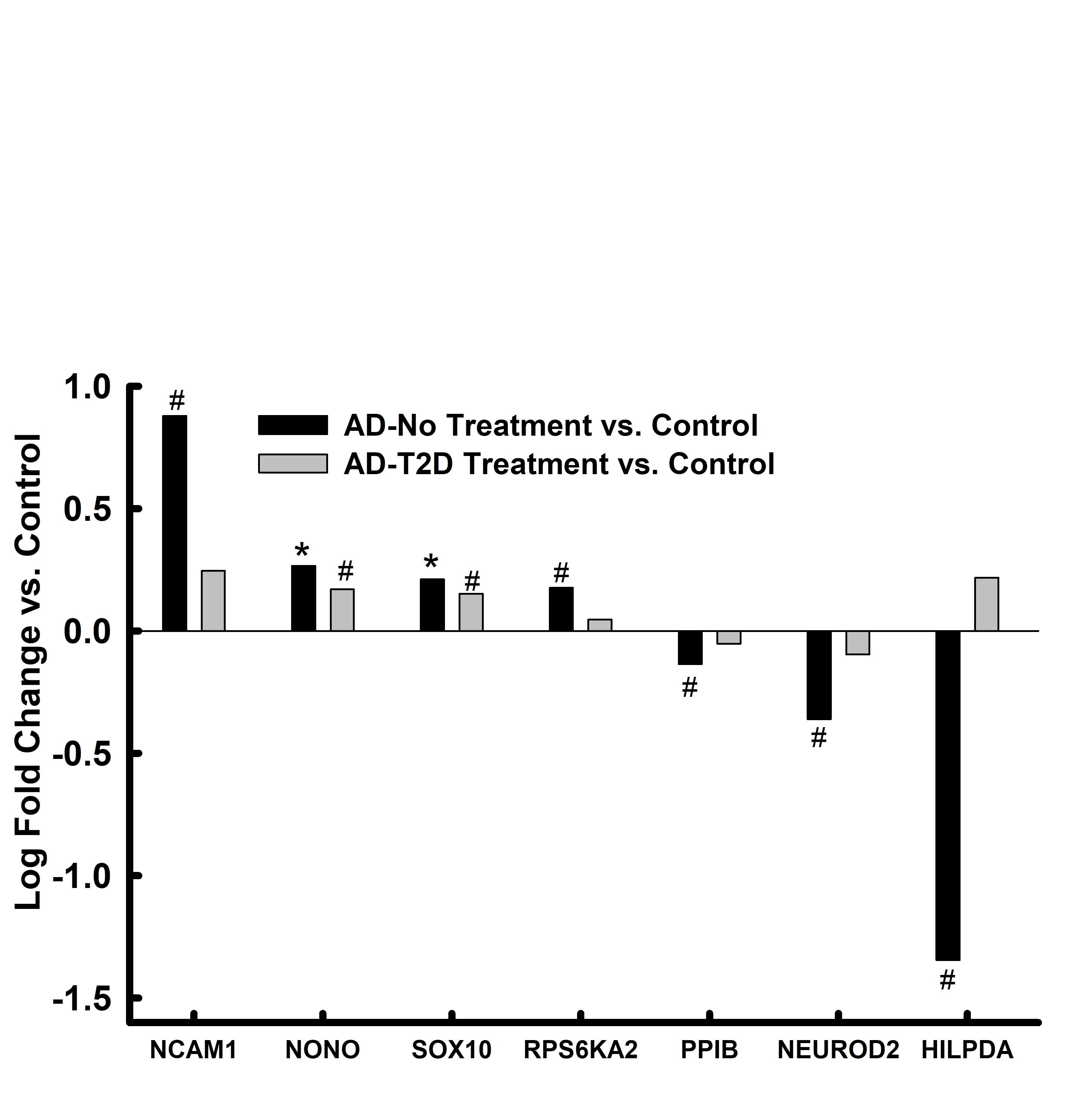

Supplement: S2 Fig — Values represent relative log fold change in persons with AD relative to controls and log fold change in persons with AD and T2D who had been treated with anti-diabetes agents. * = p<0.05 after FDC correction; # = p<0.05 without FDR correction. (TIF) [file pone.0206547.s004.TIF]
